# Effect of extracted phycocyanin by-products as a synbiotic supplement on the production performance and intestinal morphology of broilers

**DOI:** 10.14202/vetworld.2025.52-59

**Published:** 2025-01-09

**Authors:** Kannikar Hamprakorn, Buaream Maneewan, Wantamas Jantasin, Mohd Nizam Lani, Tossapol Moonmanee, Julakorn Panatuk

**Affiliations:** 1 Faculty of Animal Science and Technology, Maejo University, Chiang Mai 50290, Thailand; 2 Faculty of Fisheries and Food Science, Universiti Malaysia Terengganu, 21030 Kuala Nerus, Terengganu, Malaysia; 3 Department of Animal and Aquatic Sciences, Faculty of Agriculture, Chiang Mai University, Chiang Mai 50200, Thailand

**Keywords:** broiler, by-products, production, *Spirulina platensis*, symbiotic

## Abstract

**Background and Aim:**

The extracted phycocyanin by-products retain nutritional value, including proteins, polysaccharides, and bioactive compounds, which have the potential as feed supplements in broiler production. This study aimed to evaluate the effect of by-products acquired during phycocyanin extraction, which is used as a novel synbiotic supplement, on the production performance and intestinal health of broilers in a tropical climate.

**Materials and Methods:**

A total of 240 one-day-old male Ross 308 broilers were randomly distributed among five dietary treatment groups; they received a diet supplemented with a synbiotic product (probiotic [*Lactobacillus johnsonii*] at least 1.0 × 10^8^ colony-forming unit/mg with prebiotic [by-product of phycocyanin extraction]) at 0.000%, 0.025%, 0.050%, 0.075%, and 0.100%. We investigated the effects of dietary synbiotic supplements on the growth performance, meat quality, intestinal morphology, and cecal bacterial population of broiler chickens aged 35 days.

**Results:**

Synbiotics used as a dietary supplement did not affect the growth performance of broilers during any experimental period (p > 0.05); however, it significantly increased the redness of meat and decreased the levels of thiobarbituric acid-reactive substances on days 3 and 7 of storage (p < 0.05). Moreover, synbiotics significantly improved the height and surface area of villi in the duodenum and jejunum (p < 0.05).

**Conclusion:**

The study demonstrated that dietary supplementation with 0.1% synbiotics, incorporating a by-product of phycocyanin extraction, did not significantly influence the growth performance of broiler chickens. However, it positively affected meat quality by increasing redness and reducing lipid oxidation during storage. Additionally, synbiotic supplementation significantly enhanced intestinal health by improving the villi height and surface area in the duodenum and jejunum, highlighting its potential benefits for broiler intestinal morphology and meat quality in tropical climates. Further research is recommended to explore the mechanisms underlying these effects and their implications for long-term poultry health and productivity.

## INTRODUCTION

Over the last four decades, the importance of poultry gastrointestinal health and immunity has increased significantly [1]. Currently, probiotics and prebiotics are used in poultry farms to address the gut health concerns of chickens. Probiotics are live microorganisms that positively influence the health of the host through immunomodulation, competitive exclusion of gut pathogens, and promotion of the diversity and stability of intestinal bacteria. Lactobacilli are the most common group of lactic acid bacteria naturally occurring in the gastrointestinal tracts of poultry, where they can serve as probiotics. *Lactobacillus salivarius*, *Lactobacillus* *johnsonii*, *Lactobacillus crispatus*, *Lactobacillus reuteri*, and *Lactobacillus agilis* are the most frequently identified species in poultry [2, 3]. Prebiotics are non-digestible carbohydrates that influence the intestinal bacteria and the immunity of broiler chickens by acting as substrates required for the growth of bifidobacteria and lactic acid bacteria in the colon [4]. Prebiotics improve the survival and multiplication of probiotics by enhancing their tolerance to high temperatures, oxygen, and low pH. Combining prebiotics and probiotics has synergistic advantages in broiler production [5]. Synbiotics, a combination of prebiotic and probiotic bacteria, are gaining popularity as functional feed supplements for poultry nutrition production. Synbiotic supplements in the diet can enhance growth performance, intestinal morphology, and nutrient absorption [6].

*Spirulina platensis* stimulates bacterial growth, immune function, reproduction, and growth performance in broilers. In broilers, *Spirulina*-containing diets increase mineral absorption, protect against diarrhea, improve nutrient digestion systems, and promote antigen processing [7], inhibits the synthesis of cytokines interleukin (IL)-1, IL-6, and tumor necrosis factor-gamma [8], and induces microbial killing [8]. A carbohydrate content of approximately 13% and a crude protein (CP) concentration of 60%–70% were detected in *S. platensis* [9]. The structural proteins in *Spirulina* include phycobiliproteins, a group of water-soluble fluorescent proteins [10]. C-phycocyanin is a major component of the phycobiliprotein family, with a high aggregate value and ability to produce. Phycocyanin exhibits antiviral, anti-inflammatory, and antitumor activities and is used as a fluorescent marker in biomedical research [11]. However, phycocyanin isolated from *Spirulina* is still a by-product. The by-product from the phycocyanin extraction process is expected to retain the prebiotic function.

Based on our hypothesis on synbiotic supplementation to improve the production and gut health in broilers [5, 6], this study aimed to investigate whether a synbiotic (a combination of a by-product of phycocyanin extraction, which served as a prebiotic and probiotic [*L. johnsonii*]) dietary supplementation affects the growth performance, meat quality, intestinal morphology, and cecal coliform population of broilers.

## MATERIALS AND METHODS

### Ethical approval

All experimental procedures were approved by the Animal Care and Use Committee of Maejo University (MACUC006A/2564). All experiments were performed in accordance with the guidelines of the International Guiding Principles for Animal Research and Welfare.

### Study period and location

This study was conducted from April 2022 to June 2022 at the Poultry Farm, Meat Laboratory, Molecular Biology Laboratory, and Histology Laboratory of the Faculty of Animal Science and Technology, Maejo University, Thailand.

### Experimental animals

A total of 240 one-day-old male broilers for stable production performances (Ross 308 strain from a commercial hatchery) were used in the present study. The acclimation period was 7 days. The birds were weighed and randomly assigned to five groups, each comprising four replicates (12 birds per replicate), according to a completely randomized design (CRD). The experimental diet of these broilers consisted of a basal diet supplemented with different synbiotic concentrations (0%, 0.025%, 0.050%, 0.075%, and 0.100%). In this study, a synbiotic product was prepared by combining the probiotic (*L. johnsonii* isolated and characterized at the Faculty of Science, Maejo University, Thailand; at least 1.0 × 10^8^ colony-forming unit [CFU]/g) with the prebiotic oligosaccharide and cell wall fragments derived from the by-product of phycocyanin extraction from *S. platensis*. Birds were raised in identical-sized cages (2.5 m × 2 m) in a deep-litter system using rice husks as bedding material. Experimental diets and fresh water were provided daily (*ad libitum*). Basal diets were provided following recommendations for Ross 308 broilers. Diets were divided into three phases: starter (23% CP and 29 metabolizable energy [ME] kJ/kg for days 1–14), grower (13% CP and 29 ME kJ/kg for days 15–28), and finisher (19% CP and 29 ME kJ/kg for days 29–35). During the experiment, birds were maintained at approximately 31°C. The research institute implemented a routine vaccination program.

### Data collection and sampling procedures

The birds were weighed at the initiation of the experiment. Correspondingly, the body weight of individual replicates was recorded weekly before providing feed and water. Body weight gain and feed intake were calculated. The feed conversion ratio was calculated by dividing the feed intake by the body weight gain. At the end of the experimental period, four birds from each treatment group were randomly selected for blood sample collection. Blood samples were collected from jugular veins. Approximately 6 mL aliquots of blood from each bird were collected in a blood tube containing an anticoagulant (Ethylenediaminetetraacetic acid [Hebel Xinle Sci & Tech®, Hebei, China]) and in a blood tube without an anticoagulant and used in the biochemical analysis. A total of eight 35-day-old chickens from each group were randomly selected and slaughtered via cervical dislocation. The carcasses were dissected immediately to assess their characteristics and the percentage of edible meat.


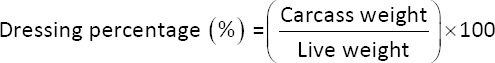



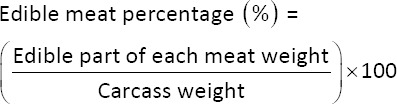


Meat samples were collected from the breast muscles of birds in each experimental group (n = 8/treatment group). The pH of the breast muscle was estimated using a pH meter (HI99163 Hanna Instruments, Padua, Italy) 45 min (pH_45 min_) and 24 h (pH_24 h_) postmortem. Meat color values were measured using the International Commission on Illumination Lab* color space (CIELAB) method, and lightness (L*), redness (a*), and yellowness (b*) were assessed using the CR-400 Chroma Meter colorimeter (Konica Minolta Holding Inc., Tokyo, Japan). The chroma (C*) and hue angle (h°) were calculated based on the a* and b* color coordinates [12] using the following equations:


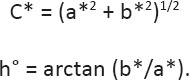


The water-holding capacity was calculated as the percentage (%) of the water weight loss (boil loss, drip loss, and freeze loss) according to the method described by Honikel [13]. The shear force of boiled meat was analyzed using the Warner-Bratzler shear force (Instron Model 3433 Universal test machine, USA).

Lipid oxidation in the breast meat was assessed by measuring the levels of 2-thio-barbituric acid-reactive substances (TBARS) [14]. The absorbance of the clear supernatant was measured in duplicates at 532 nm using an ultraviolet (UV)-visible spectrophotometer (UV-1900i, Shimadzu, Japan). TBARS values were calculated as milligrams of malondialdehyde (MDA) per kg of meat sample (mg MDA/kg meat) using a molar extinction coefficient (156,000 M^-1^/cm) [12] using the following equations:

TBARS number (mg MDA/kg) = sample A532 × [(1M TBA chromagen)/156,000] × [(1 mol/L/M) × (0.030 L/5 g meat) × (72.07 g MDA/mol MDA) × (1000 mg/g) × (1000 g/kg)].

### Intestinal morphology and cecal microbial populations

For morphometric measurements, small intestinal samples were collected from different parts of the small intestine of the birds in each experimental group (n = 8/group). An image analyzer (NIS-Elements [Nikon®, Tokyo, Japan]) was used to measure villus heights and crypt depths in histological sections. The crypt depth was defined as the depth of invagination between two adjacent villi, and the length from the tip of the villus to the villus-crypt junction was considered the villus height [15].

The cecal contents were randomly collected from each experimental group (n = 4/group) and immediately diluted 10-fold (10% w/v) using a sterile 0.9% NaCl solution. The samples were serially diluted from 10^-1^ to 10^-7^, and viable counts of *Lactobacillus* spp., *Escherichia coli*, and *Salmonella* were determined using appropriate dilutions and agar media. In this experiment, deMan Rogosa and Sharpe agar (Merck®), Eosin Methylene Blue agar (Merck®), and *Salmonella Shigella* agar (Merck®) were used to detect *Lactobacillus* spp., *E. coli*, and *Salmonella* spp. The number of bacterial CFUs was calculated and expressed as log CFU/g of fresh samples.

### Statistical analysis

The experiment was conducted with a CRD. The experimental unit of growth performance was the birds in a single cage (n = 4 with 12 birds per cage). Meat quality data were obtained from a group of eight birds considered as an experimental unit. All data were statistically analyzed using a one-way analysis of variance in Statistical Analysis System v.9.4 (SAS Inst. Inc., Cary, NC, USA). Furthermore, Duncan’s multiple range test was performed in cases of significant differences (p < 0.05) and tendencies to differ (0.05 < p ≤ 0.10).

## RESULTS

### Growth performance and carcass characteristics

The effects of synbiotic dietary supplementation on broiler growth performance at various stages are presented in Table 1. Initial body weight, weight gain, feed intake, feed conversion ratio, and final body weight did not differ significantly between groups. No significant differences were detected in the carcass characteristics (Table 2) among the experimental groups (p > 0.05).

**Table 1:** Effects of dietary synbiotic on the performance parameters of broiler chickens.

Parameters^1^	Synbiotic supplementation level (%)					SEM	Significance

	0.000	0.025	0.050	0.075	0.100		
Initial BW (g)	43.86	43.89	43.85	43.89	43.84	0.032	ns
Final BW (g)	1707.42	1664.77	1690.83	1710.67	1720.42	9.997	ns
Starter (0–14 days)							
BWG (g/bird)	262.81	258.61	254.07	264.86	265.75	3.857	ns
FI (g/bird)	459.69	465.89	446.96	456.63	475.65	5.279	ns
FCR	1.77	1.88	1.80	1.74	1.76	0.028	ns
Grower (15–28 days)							
BWG (g/bird)	778.01	710.49	748.75	768.42	799.58	13.367	ns
FI (g/bird)	1,406.19	1,362.20	1,322.59	1,349.11	1,373.72	21.554	ns
FCR	1.90	2.02	1.83	1.80	1.75	0.051	ns
Finisher (28–35 days)							
BWG (g/bird)	622.75	651.78	644.17	633.50	611.25	12.969	ns
FI (g/bird)	1,329.19	1,373.91	1,340.77	1,329.37	1,275.76	22.359	ns
FCR	2.16	2.12	2.09	2.10	2.09	0.038	ns
All periods (0–35 days)							
BWG (g/bird)	1,663.56	1,620.87	1,646.99	1,666.78	1,676.58	9.938	ns
FI (g/bird)	3,195.07	3,202.00	3,110.32	3,135.11	3,125.13	25.347	ns
FCR	1.86	1.92	1.82	1.80	1.80	0.022	ns

1 Data are expressed as mean, n=4 cages per experimental group, SEM=Standard error of the mean, ns=No significant difference, BW=Body weight, BWG=Body weight gain, FI=Feed intake, FCR=Feed conversion ratio

**Table 2:** Effects of dietary synbiotic on carcass characteristics of broiler chickens.

Carcass characteristics^1^	Synbiotic supplementation level (%)					SEM	Significance
	0.000	0.025	0.050	0.075	0.100		
Live weight (g)	1709.37	1672.58	1700.73	1716.09	1729.03	13.417	ns
(As % of live weight)							
Dressed carcass	93.17	93.18	92.53	93.12	93.12	0.179	ns
Eviscerated carcass	73.69	73.94	73.49	73.69	73.76	0.184	ns
Abdominal fat	0.46	0.40	0.50	0.41	0.38	0.020	ns
Edible meat (as % of eviscerated carcass)							
Drumsticks	10.91	10.81	10.67	10.98	11.11	0.071	ns
Thighs	13.12	12.98	12.91	12.87	12.96	0.083	ns
Wings	8.31	8.25	8.38	8.42	8.46	0.053	ns
Breast	19.66	19.71	20.13	20.35	19.85	0.196	ns

1 Data are expressed as means, n=16 birds/experimental group. SEM=Standard error of the mean, ns=No significant difference

### Meat quality

Table 3 shows the effects of synbiotic supplementation on the breast meat samples. The pH_45 min_ value was significantly higher in the control group (5.67) than in the group treated with 0.1% synbiotic (5.17; p < 0.05). Synbiotic dietary supplementation did not affect lightness (L*), yellowness (b*), and chroma, measured at 45 min postmortem, pH_24 h_, water-holding capacity, and shear force. However, the control group exhibited a significantly lower redness (a*) value (13.0) and higher hue angle (39.99) than the synbiotic groups. The yellowness (b*) value was higher in the 0.025% synbiotic group (12.29) than in the control group (11.59; p < 0.05). The TBARS values on day 0 were not affected by the group. The control group exhibited significantly higher TBARS values on day 3 (0.6835 mg MDA/kg; p < 0.05) and 7 (1.0103 mg MDA/kg; p < 0.01) than the synbiotic groups. The gross energy, dry matter, and crude fat contents were not significantly different among the groups. Nevertheless, the CP content of the control group (85.75% dry matter) was lower than that of the synbiotic groups (p < 0.05).

**Table 3:** Effects of dietary synbiotic on the breast meat quality of broiler chickens.

Parameters^1^	Synbiotic supplementation level (%)					SEM	Significance

	0.000	0.025	0.050	0.075	0.100		
pH_45 min_	5.67^a^	5.63^ab^	5.67^a^	5.61^ab^	5.57^b^	0.012	*
pH_24 h_	5.73	5.76	5.71	5.65	5.66	0.015	ns
Color_45 min_							
Lightness (L*)	50.30	48.73	50.52	49.33	50.10	0.459	ns
Redness (a*)	13.0^b^	15.28^a^	15.49^a^	15.77^a^	15.70^a^	0.296	*
Yellowness (b*)	10.95	10.87	11.17	9.32	11.43	0.255	ns
Chroma	17.06	18.82	19.11	18.38	19.46	0.296	ns
Hue angle	39.99^a^	35.58^a^	35.74^a^	30.57^b^	35.99^a^	0.787	*
Color_24 h_							
Lightness (L*)	53.27	54.00	53.51	54.95	55.33	0.411	ns
Redness (a*)	15.80	17.76	18.13	17.96	18.32	0.324	ns
Yellowness (b*)	11.59^b^	12.29^a^	11.69^ab^	11.49^b^	11.70^ab^	0.285	*
Chroma	19.65	21.66	21.59	21.35	21.79	0.307	ns
Hue angle	36.53	35.00	32.83	32.52	32.51	0.656	ns
Water-holding capacity (%)							
Boil loss (%)	16.76	16.37	15.06	15.90	16.04	0.337	ns
Drip loss (%)	6.56	6.21	5.72	5.09	5.61	0.203	ns
Freeze loss (%)	6.00	4.92	5.42	4.92	5.16	0.221	ns
Shear force value (kg)	1.663	1.280	1.349	1.329	1.239	0.071	ns
TBARS (mg MDA/kg)							
0 days	0.2360	0.2352	0.2537	0.2513	0.2293	0.009	ns
3 days	0.6835^a^	0.3853^b^	0.3910^b^	0.3813^b^	0.3770^b^	0.037	*
7 days	1.0103^a^	0.4573^b^	0.4307^b^	0.4410^b^	0.4315^b^	0.046	***
Chemical composition^2^							
Gross energy (cal/g)	5116.95	5156.45	5194.45	5169.50	5159.10	9.551	ns
DM (%)	93.44	93.38	93.52	93.34	93.55	0.183	ns
Crude protein (% DM)	85.75^b^	88.23^a^	88.67^a^	88.91^a^	88.74^a^	0.429	*
Crude fat (% DM)	6.67	6.82	6.78	6.79	6.82	0.035	ns

1 Data are expressed as means; n=8 birds/experimental group, ^a,b^Within a row, the mean values with different superscript letters differ significantly (p < 0.05). SEM=Standard error of the mean; ns, no significant difference, DM=Dry matter, TBARS=Thio-barbituric acid reactive substances, MDA=Malondialdehyde.

* p < 0.05,

*** p < 0.001

### Intestinal morphology and cecal coliform population

**Figure 1:**
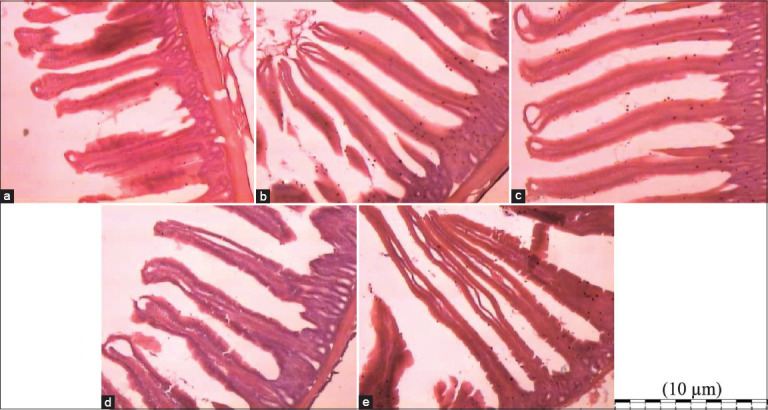
Histological picture of the small intestine (Jejunum) (40×). (a) Control group fed with no synbiotic, (b) 0.025 group fed with synbiotic (0.025%), (c) 0.050 group fed with symbiotic (0.025%), (d) 0.075 group fed with synbiotic (0.075%), (e) 0.100 group fed with synbiotic (0.100%).

The synbiotic group did not affect villi and crypt depth in the duodenum and jejunum (p > 0.05; Table 4 and Figure 1). However, shorter villus heights and crypt depths in the ileum were detected in the synbiotic group than in the control group (p < 0.05). The villus surface area was larger in the synbiotic group than in the control group (p < 0.05). *Lactobacillus* populations were enhanced by synbiotic supplementation. Lactobacilli were more abundant (8.939 log CFU/g) in the 0.1% synbiotic group than in the control group (7.011 log CFU/g). Nonetheless, the *E. coli* population decreased in the synbiotic supplementation group compared with the control group (6.208 log CFU/g). No *Salmonella* spp. was detected in this study.

**Table 4:** Effects of dietary synbiotic on the intestinal morphology and cecal coliform population (log CFU/g) of broiler chickens.

Parameters^1^	Synbiotic supplementation level (%)					SEM	Significance

	0.000	0.025	0.050	0.075	0.100		
Duodenum							
Villus height (mm)	18.95	18.91	20.69	19.70	20.64	0.318	ns
Crypt depth (mm)	2.33	2.11	2.11	2.09	2.22	0.046	ns
Villus surface area (mm^2^)	26.28^b^	30.03^ab^	33.08^a^	34.11^a^	33.79^a^	0.832	***
Jejunum							
Villus height (mm)	10.03	13.97	14.77	14.73	15.32	0.271	ns
Crypt depth (mm)	2.36	2.35	2.20	2.30	2.22	0.053	ns
Villus surface area (mm^2^)	18.41^b^	20.46^b^	25.35^a^	26.10^a^	25.12^a^	0.850	***
Ileum							
Villus height (mm)	9.48^a^	8.17^b^	8.39^ab^	7.65^b^	7.99^b^	0.201	*
Crypt depth (mm)	2.23^a^	1.62^bc^	1.74^b^	1.53^c^	1.52^c^	0.050	***
Villus surface area (mm^2^)	13.86	15.73	17.25	15.25	17.31	0.580	ns
Cecal coliform population (log CFU/g)							
*Lactobacillus* spp.	7.011^b^	8.682^a^	8.891^a^	8.813^a^	8.939^a^	0.228	*
*Escherichia* *coli*	6.208^a^	5.837^ab^	5.551^b^	5.439^b^	5.323^b^	0.101	*

1 Data are expressed as mean n=4 birds/experimental group, ^a,b^Within a row, the mean values with different superscript letters differ significantly (p < 0.05). SEM=Standard error of the mean, ns=No significant difference.

* p < 0.05,

*** p < 0.001

## DISCUSSION

The results of this study show no significant effects of synbiotic-supplemented feed on growth performance or carcass characteristics. This result is consistent with that reported by Bogucka *et al*. [16]; this report reflected that synbiotic supplementation did not affect broiler chicken performance parameters [2]. In contrast, other studies reported by Kridtayopas et al. [6], Amerah et al. [17], Ghasemi-Sadabadi et al. [18], Hassanpour et al. [19] that probiotic and/or prebiotic supplementation could improve growth performance without affecting the carcass characteristics of broiler chickens [6, 17–19], inhibit resistant bacteria, such as *E. coli* and *Salmonella*, and increase nutrient digestibility [6]. The variations in the effects of probiotics and synbiotics, which were reflected in previous studies by Mookiah *et al*. [20] and Zammit and Park [21], are potentially attributed to the differences in bacterial strains and the concentrations of dietary supplements. However, *Spirulina* used as a prebiotic in broiler chicken diets increases body weight and promotes weight gain on days 7, 14, and 28 [7] by the promoted growth of the *Lactobacillus* population and improved absorption of dietary micronutrients [22]. Moreover, some studies by El-Abd *et al*. [23] found that dietary *A. platensis* phycocyanin demonstrates a beneficial effect on body weight, weight gain, and feed consumption ratio in broiler chickens.

The physicochemical properties of meat, including pH, water-holding capacity, and protein content, are critical in determining effective storage and processing options [24]. In this study, the synbiotic-treated group exhibited a considerably lower pH in the breast muscle than the control group. Synbiotic supplementation considerably increased redness (a*) in the breast and thigh muscles 45 min postmortem. *Spirulina* contains naturally occurring carotenoid-like pigments that can serve as the source of color in fish, eggs, and chicken [25]. Products derived from *Spirulina* can support the production of chicken meat with increased yellowness and redness through the enhanced deposition of pigments [26]. The ultimate pH of muscle significantly contributes to meat quality, which is reflected by its softness, color, and storage life [27]. The TBARS evaluation is the most extensively used to study MDA concentration in the body, which is induced by diminished antioxidant protection against free radicals [28]. Lipid oxidation is one of the most important causes of reduced food quality, associated with the appearance of off-odors and flavors [24]. This study detected reduced TBARS values in the synbiotic group on days 3 and 7 of storage compared to the control counterpart. Similarly, Dev *et al*. [11] determined that synbiotic supplements tend to decrease the TBARS, peroxide, and free fatty acid values in chicken meat. Various probiotics and prebiotics reduced TBARS levels in chicken meat, reflecting reduced lipid oxidation [29]. Water-holding capacity and shear force tended to decrease in the synbiotic supplement group. However, Dev *et al*. [11] reported that the effect of dietary supplements on meat quality significantly depends on the probiotic strain.

Moreover, previous studies by Acharya *et al*. [30] and Das *et al*. [31] have shown that synbiotics selectively reduce pathogen populations and increase beneficial microorganisms and immunity, which can reduce mortality and increase the growth of animals. Supplementing with the product obtained from *S. platensis* can promote the digestion of feed by modulating the histology of duodenum, jejunum, and ileum [32]. In this study, synbiotic supplementation significantly affected intestinal morphology through improved villus height and surface area [19, 30]. Probiotics and prebiotics enhanced intestinal crypt cell proliferation, villus height, and the surface area of the villi. The synbiotic supplementation influenced intestinal morphology, particularly in the jejunum and ileum, and significantly affected the cecal coliform population by promoting *Lactobacillus* populations and decreasing *E. coli* populations. The gut microbiota is essential for animal productivity; it influences feed digestion and controls animal health through interactions with the host to prevent diseases [33]. Moreover, different *Lactobacillus* strains have been shown to enhance growth and immunity in broilers and inhibit the growth of pathogenic gut bacteria [2, 20]. Synbiotic supplementation increases the abundance of lactic acid bacteria in the digestive tract and improves immunity, nutrient digestibility, and feed utilization; furthermore, it reduces mortality and production costs. Some bacteria may recognize binding sites on prebiotics or synbiotics in the intestinal mucosa [34], which potentially reduces harmful bacterial colonization (e.g., *E. coli*) in the intestine [33]. Moreover, an increase in nutrient absorption may directly influence the repair of the intestinal mucosa by increasing the height of the villi.

## CONCLUSION

The study revealed that dietary supplementation with 0.1% synbiotics derived from phycocyanin by-products significantly improved meat quality and reduced lipid oxidation in broiler chickens while enhancing intestinal morphology through increased villus surface area and promoting beneficial *Lactobacillus* populations. The findings highlight the potential of these synbiotics as a natural alternative to conventional feed supplements, particularly for improving poultry production in tropical climates. However, the study’s limitation lies in its focus on a single strain of probiotics (*L. johnsonii*) and its relatively short experimental duration, which may not fully capture long-term impacts or the effects of varying probiotic strains. Additionally, growth performance remained unaffected, which suggests that synbiotic supplementation may not directly influence overall production efficiency. Future research should explore the use of diverse probiotic strains, conduct long-term studies across different environmental and management systems, and assess the economic feasibility of incorporating such by-products into broader poultry production systems to optimize both performance and sustainability outcomes.

## AUTHORS’ CONTRIBUTIONS

KH: Research implementation and statistical analysis and drafted and revised the manuscript. BM, WJ, MNL, TM, and JP: Designed and conducted the study. JP: Supervised the study and critically revised the manuscript for important intellectual content. All authors have read and approved the final version of the manuscript.
